# Characteristics of randomized controlled trials of yoga: a bibliometric analysis

**DOI:** 10.1186/1472-6882-14-328

**Published:** 2014-09-02

**Authors:** Holger Cramer, Romy Lauche, Gustav Dobos

**Affiliations:** Department of Internal and Integrative Medicine, Kliniken Essen-Mitte, Faculty of Medicine, University of Duisburg-Essen, Am Deimelsberg 34a, 45276 Essen, Germany

**Keywords:** Yoga, Complementary therapies, Randomized controlled trials, Bibliometrics, Review

## Abstract

**Background:**

A growing number of randomized controlled trials (RCTs) have investigated the therapeutic value of yoga interventions. This bibliometric analysis aimed to provide a comprehensive review of the characteristics of the totality of available randomized yoga trials.

**Methods:**

All RCTs of yoga were eligible. Medline/PubMed, Scopus, the Cochrane Library, IndMED, and the tables of content of yoga specialty journals not listed in medical databases were screened through February 2014. Bibliometric data, data on participants, and intervention were extracted and analyzed descriptively.

**Results:**

Published between 1975 and 2014, a total of 366 papers were included, reporting 312 RCTs from 23 different countries with 22,548 participants. The median study sample size was 59 (range 8–410, interquartile range = 31, 93). Two hundred sixty-four RCTs (84.6%) were conducted with adults, 105 (33.7%) with older adults and 31 (9.9%) with children. Eighty-four RCTs (26.9%) were conducted with healthy participants. Other trials enrolled patients with one of 63 varied medical conditions; the most common being breast cancer (17 RCTs, 5.4%), depression (14 RCTs, 4.5%), asthma (14 RCTs, 4.5%) and type 2 diabetes mellitus (13 RCTs, 4.2%). Whilst 119 RCTs (38.1%) did not define the style of yoga used, 35 RCTs (11.2%) used Hatha yoga and 30 RCTs (9.6%) yoga breathing. The remaining 128 RCTs (41.0%) used 46 varied yoga styles, with a median intervention length of 9 weeks (range 1 day to 1 year; interquartile range = 5, 12). Two hundred and forty-four RCTs (78.2%) used yoga postures, 232 RCTs (74.4%) used breath control, 153 RCTs (49.0%) used meditation and 32 RCTs (10.3%) used philosophy lectures. One hundred and seventy-four RCTs (55.6%) compared yoga with no specific treatment; 21 varied control interventions were used in the remaining RCTs.

**Conclusions:**

This bibliometric analysis presents the most complete up-to-date overview on published randomized yoga trials. While the available research evidence is sparse for most conditions, there was a marked increase in published RCTs in recent years.

**Electronic supplementary material:**

The online version of this article (doi:10.1186/1472-6882-14-328) contains supplementary material, which is available to authorized users.

## Background

Yoga is rooted in Indian philosophy and has been a part of traditional Indian spiritual practice for millennia [1]. The Indian sage Patañjali prescribed adherence to eight limbs of yoga, aimed at quieting one’s mind to achieve the union of mind, body and spirit that is traditional yoga’s ultimate goal. These limbs include ‘*Yama’ and ‘Niyama’* (a code of conduct for an ethical lifestyle), ‘*Asana’* (physical postures), ‘*Pranayama’* (breath control), ‘*Pratyahara’* (withdrawal of the senses from external objects to increase self-awareness), ‘*Dharana’* (concentration), ‘*Dhyana’* (meditation) and ‘*Samadhi’* (oneness with the object of meditation) [1, 2]. Regardless of its spiritual origins, yoga has become a popular route to physical and mental well-being [1, 2] and has been adapted for use in complementary and alternative medicine in Western society [3]. In the latter setting, yoga is most often associated with physical postures, breath control and meditation; and different yoga schools have emerged that put varying focus on physical and mental practices [2].

Worldwide, it is estimated that yoga is regularly practiced by about 30 million people [4]. Yoga is gaining increased popularity as a therapeutic practice; nearly 14 million Americans (6.1% of the United States of America’s population) reported that yoga had been recommended to them by a physician or therapist [5]. Indeed, about half of American yoga practitioners (more than 13 million people) reported starting practice explicitly to improve their health [6, 7]. In the United Kingdom, yoga is even promoted by the National Health Service as a safe and effective approach, in health and illness, for people of all ages [8].

Yoga’s therapeutic potential has been explored in a growing number of randomized controlled trials (RCTs) to date [9]. In order to inform practitioners, therapists and patients about the therapeutic value of yoga in a specific condition, it is important to consolidate knowledge on the available research evidence. The aim of this bibliometric analysis was to provide a comprehensive review of the characteristics of the totality of available randomized yoga trials.

## Methods

Where applicable, this bibliometric analysis is reported in accordance with the PRISMA (Preferred Reporting Items for Systematic Reviews and Meta-Analyses) guidelines [10].

### Eligibility criteria

#### Types of studies

Randomized controlled trials (RCTs), cluster-randomized trials and randomized cross-over studies were eligible. No language restrictions were applied.

#### Types of participants

Studies of all types of participants were eligible. No restrictions were applied regarding socio-demographic characteristics or health status.

#### Types of interventions

Studies were eligible if they assessed the effects of yoga interventions. No restrictions were applied regarding the tradition, length, frequency or duration of the studied yoga programs. The specific yoga practices included in the intervention were not restricted as long as the intervention was based on yoga theory and/or traditional yoga practice. Eligible intervention components included yoga postures, yoga breathing techniques and meditation, and lectures on yoga philosophy and/or yoga lifestyle. Studies that allowed individual co-interventions, in addition to the intervention formally studied were deemed eligible, but those with multimodal interventions (such as mindfulness-based stress reduction or comprehensive lifestyle modification) were not, even if the latter included yoga. Studies with all types of control interventions were deemed eligible.

### Literature search methods

Four electronic databases, Medline/PubMed, Scopus, IndMED and the Cochrane Library were searched from their inception through February 12, 2014. The literature search was constructed around search terms for “yoga” and a filter for retrieving randomized controlled trials [11]. The complete search strategy for Medline/Pubmed is shown in Table 1. The reference lists of identified original articles or reviews were also searched manually for additional eligible studies. Additionally, the tables of contents of the *Journal of Yoga & Physical Therapy* and the *International Scientific Yoga Journal SENSE* were reviewed. Identified abstracts were screened independently by two review authors (RL, HC). Potentially eligible articles were then read in full by two review authors (HC, RL) to determine whether they actually met the eligibility criteria.

**Table 1:** **Search strategy for PubMed/Medline**

PubMed	
#1	Yoga [MeSH Terms]
#2	Yoga* [Title/Abstract] OR Yogic [Title/Abstract] OR Pranayam* [Title/Abstract] OR Asana* [Title/Abstract]
#3	#1 OR #2
#4	Randomized Controlled Trial [Publication Type] OR controlled clinical trial [Publication Type] OR randomized [Title/Abstract] OR placebo [Title/Abstract] OR random [Title/Abstract] OR randomly[Title/Abstract] OR trial [Title/Abstract] OR group [Title/Abstract]
#5	#3 AND #4

Asterisks (*) represent truncations (PubMed finds all terms that begin with a given text string).

### Data extraction and analysis

Bibliometric data (publication year, origin, journal of publication), data on participants (origin, sample size, gender, age, medical condition) and intervention (yoga tradition, program length, intervention components, control intervention) were extracted by the latter authors (HC,RL), using a standardized data extraction form. These data were then analyzed descriptively using SPSS® (release 20.0, IBM, Armonk, NY, USA) and Microsoft Excel (version 12.3.5, Microsoft, Redmond, WA, USA), to determine their distribution, central tendency (median) and dispersion (range, interquartile range [IQR]).

## Results

### Literature search

**Figure 1:**
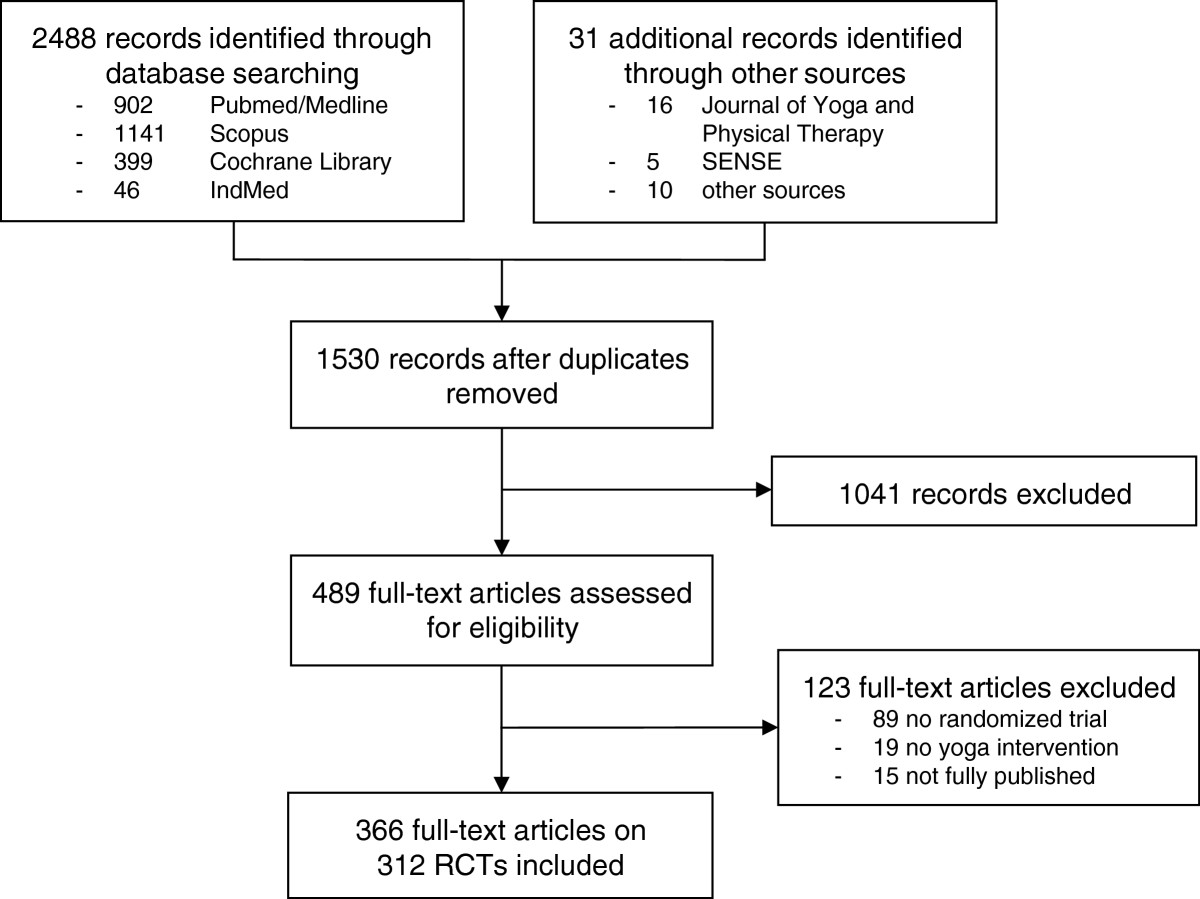
**Results of the literature search.**

A total of 2,488 records were located in the literature search, an additional 31 others being identified from other sources. Duplicate records were then excluded, leaving 1530 records to be screened. Of these, 1041 were excluded either because they were not randomized or because they did not include yoga as an intervention. Of the remaining 489 full-text records assessed for eligibility, 124 were excluded because either they were not fully published [12–26], were not randomized [27–115] or they did not include yoga interventions [116–134]. The final analysis was conducted on 366 full-text articles [135–500] reporting 312 RCTS with a total of 22,548 participants (Figure 1).

### Bibliometrics

**Figure 2:**
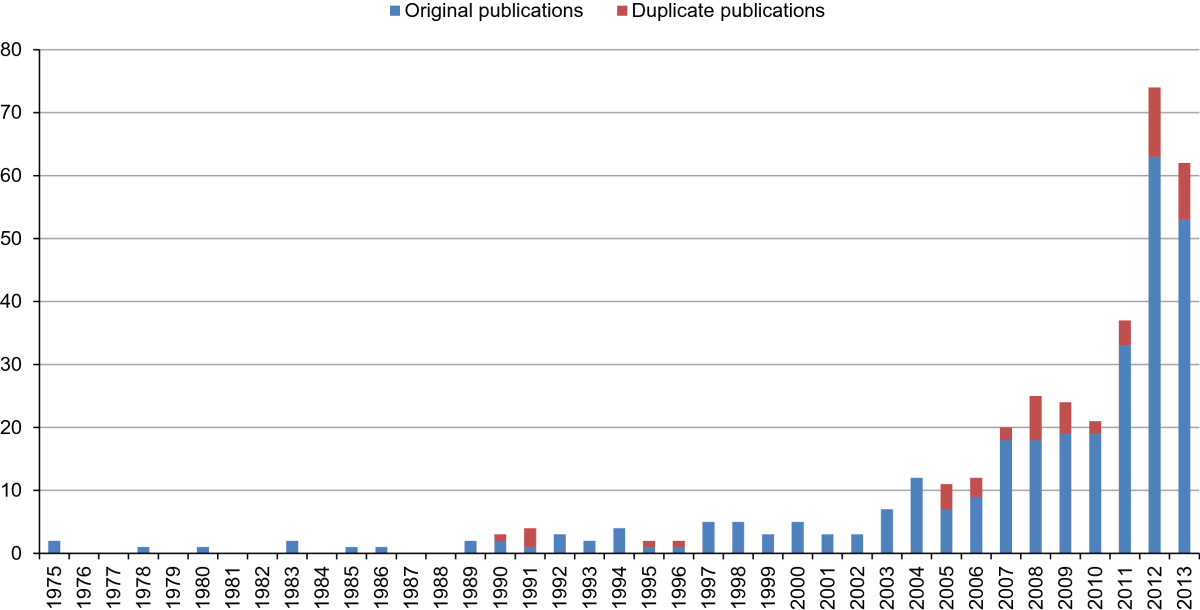
**Year of publication.** Numbers of publications between 1975 (year of first published RCT) and 2013 (last year completely covered by the literature search).

The 366 analyzed articles originated in 23 different countries, with most coming from India (k = 170, 46.4%) or the USA (k = 98, 26.8%). The two earliest pieces were published in 1975; but most (86.9%) were published post-millennial. Between 2010 and 2011, and again between 2011 and 2012, the number of articles published each year almost doubled, and remained relatively stable from 2012 to 2013 (Figure 2). Of the 366 articles identified, 54 (14.8%) proved to be duplicate publications (Figure 2); that is multiple articles reporting identical or different results on 28 already published studies; with only seven disclosing the existence of the latter. Of 170 Indian articles, only 136 were original publications, the remainder (k = 34, 20.0%) being duplicates of already published RCTs. Of the US publications, 11 pieces (11.2%) were duplicated in this way. The 366 analyzed articles appeared in 155 different journals; the most prevalent being the *International Journal of Yoga* (k = 27, 7.4%), the *Journal of Alternative and Complementary Medicine* (k = 21, 5.7%), the *Indian Journal of Physiology and Pharmacology* (k = 19, 5.2%), *Evidence-based Complementary and Alternative Medicine* (k = 9, 2.5%), *Complementary Therapies in Medicine* (k = 8, 2.2%), the *Indian Journal of Psychiatry* (k = 8, 2.2%), the *Journal of Yoga & Physical Therapy* (k = 7, 1.9%), *Alternative Therapies in Health and Medicine* (k = 6, 2.2%) and the *Indian Journal of Medical Research* (k = 6, 1.6%). Two journals published five articles, four journals published four articles, eight journals published three articles and 29 journals published two articles apiece. The remaining 147 journals each published a single paper. In total, 42 articles were published in yoga specialty journals, 58 articles in journals specialized on complementary therapies or integrative medicine, and 193 in other journals including major general medicine journals like *Lancet* (k = 2, 0.7%) [370, 429], *JAMA* (k = 1, 0.4%) [236], and *Annals of Internal Medicine* (k = 2, 0.7%) [425, 464]. Almost all articles were written in English (k = 359, 97.8%), with two each (0.5%) in Japanese and Chinese and one each (0.3%) in German, Polish, Portuguese and Farsi.

### Participants

**Figure 3:**
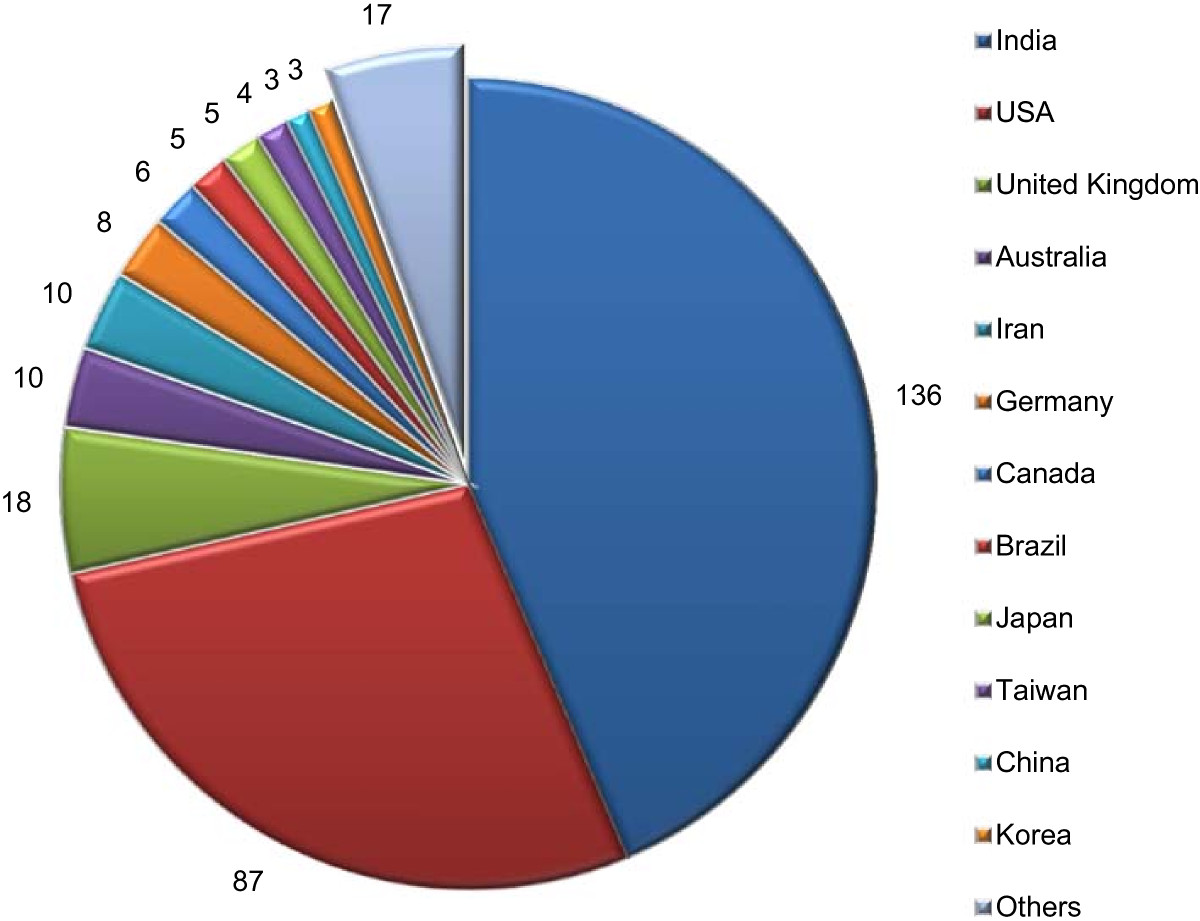
**Origin of RCTs.** Number of RCTs (not publications) classified according to country in descending order. Others = countries with just 1 RCT.

A total of 22,548 participants, from 23 different countries and five continents (Asia, North America, South America, Europe and Australia), took part in the 312 included RCTs (Figure 3). Study sample sizes ranged from 8–410 (median 59, IQR = 31, 93).

**Figure 4:**
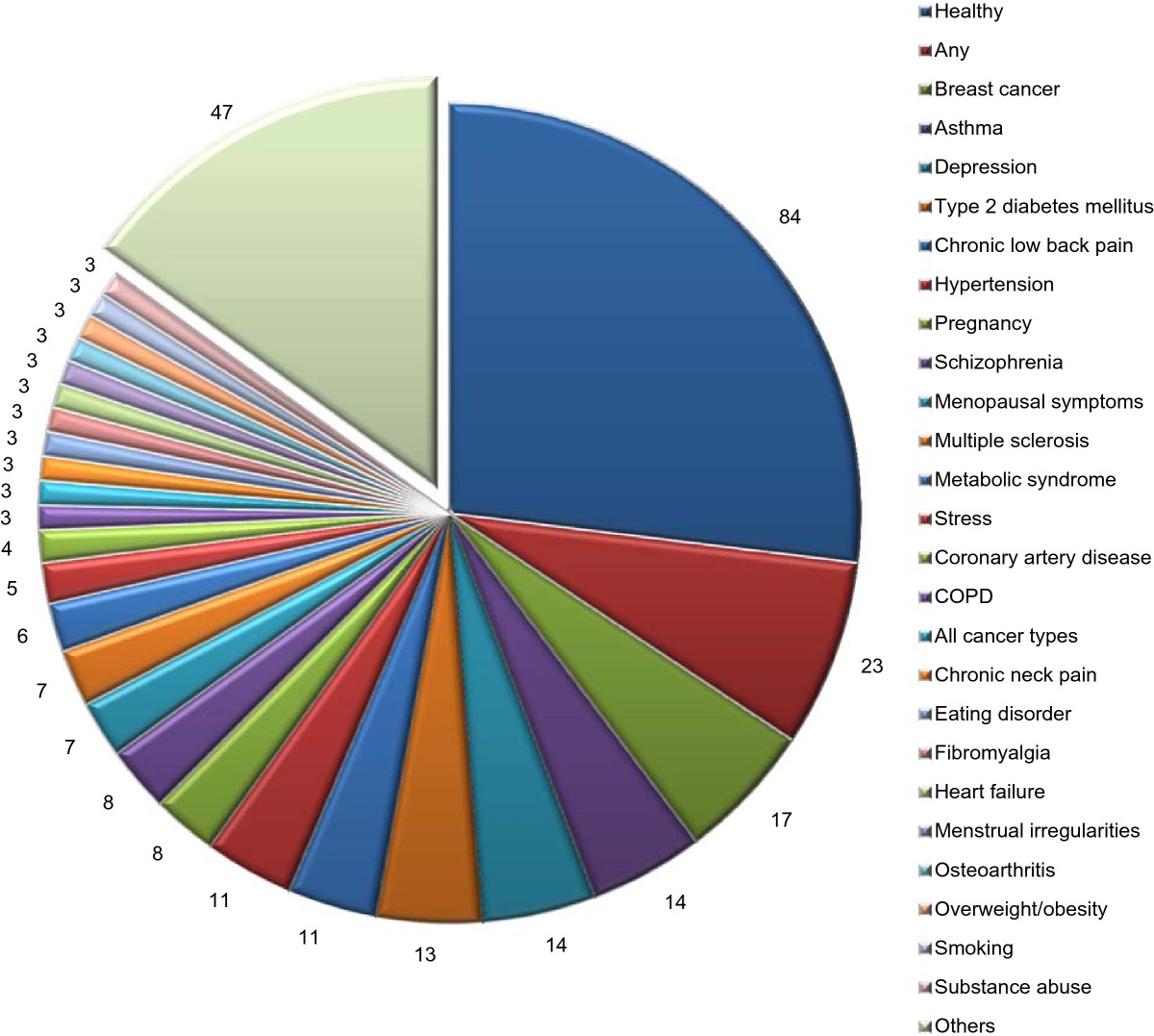
**Medical conditions.** Number of RCTs classified according to condition in descending order. Others = conditions that were studied in just 1 or 2 RCT.

The proportion of female participants ranged from 0% (30 RCTs, 9.6%) to 100% (81 RCTs, 26.1%), with a median of 67.0% (IQR = 41.3%, 100.0%). Twenty-eight RCTs (9.0%) did not report the proportion of female participants. Most studies (264 RCTs, 84.6%) included adults (aged 18–64 years), with 169 of these enrolling only adults (54.2%). Children and/or adolescents (<18 years) were included in 31 RCTs (9.9%), with 26 (8.3%) of these enrolling only children. Older adults (≥65 years) participated in 105 RCTs (33.7%), with 14 (4.5%) of these enrolling only older adults.With regard to participants’ health, 84 RCTs (26.9%) were conducted only with healthy individuals. A further 23 RCTs (7.4%) included participants from the general population, or from subpopulations such as students or employees, without any specified medical conditions as inclusion criteria. The remaining trials included participants with 63 varied predefined medical or mental health conditions (Figure 4). The most common disease categories were neurological or psychiatric disorders with 59 RCTs on 21 different conditions; spinal pain or rheumatologic diseases (7 conditions, 25 RCTs); and cardiovascular or metabolic diseases (7 conditions, 39 RCTs).

### Interventions

**Figure 5:**
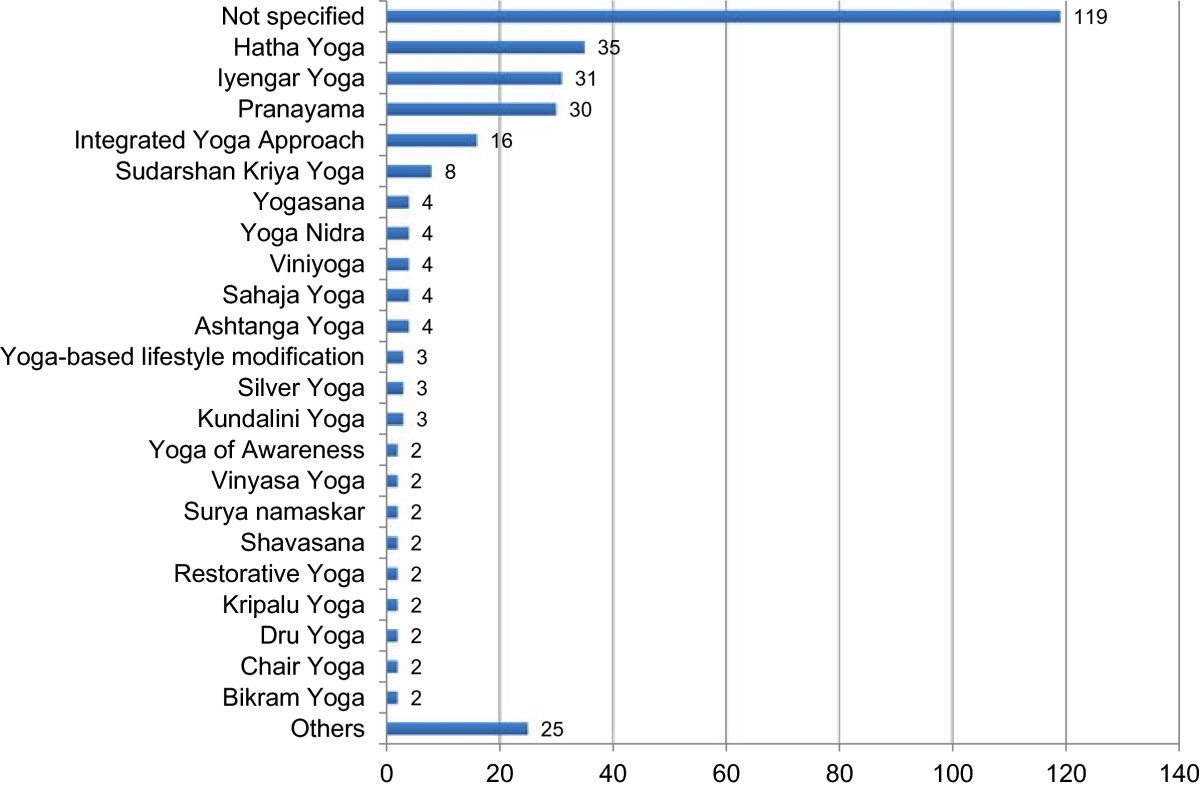
**Yoga styles.** Number of RCTs classified according to yoga style uses in the intervention. Others = yoga styles that were used in just 1 RCT.

**Figure 6:**
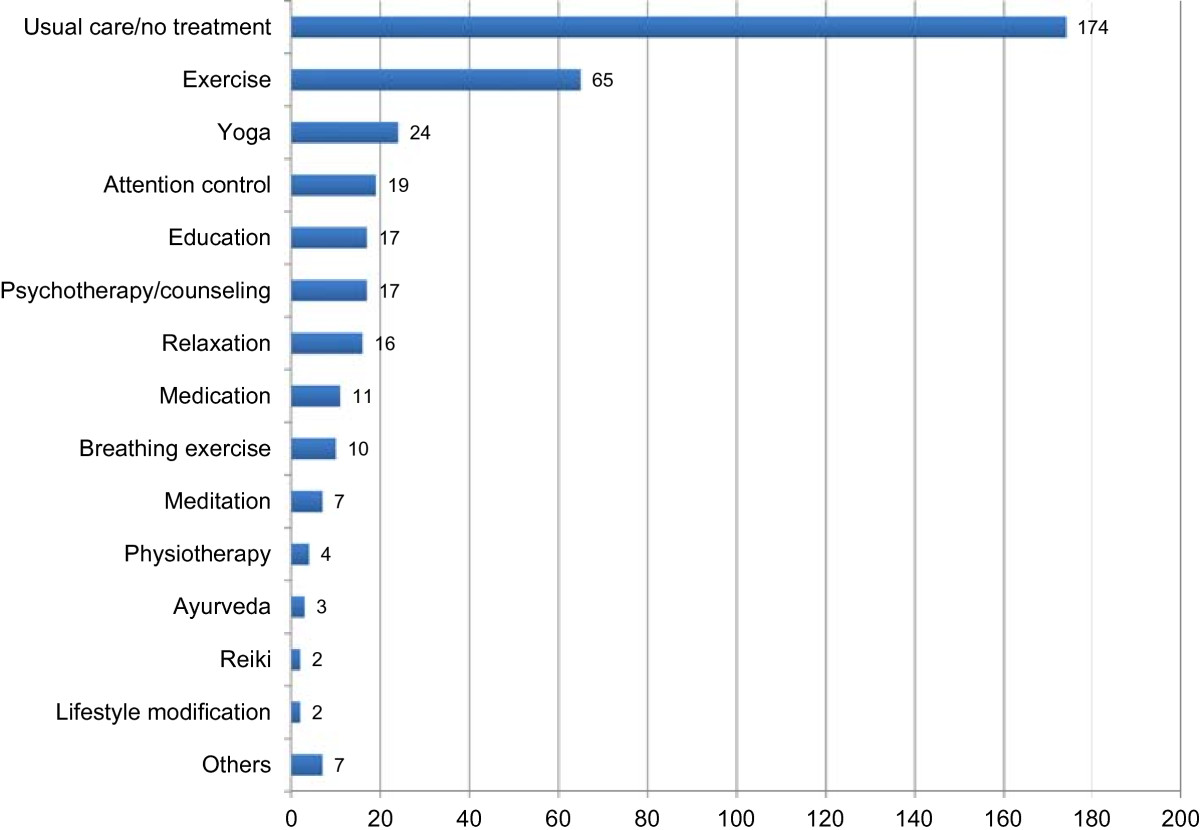
**Control interventions.** Number of RCTs classified according to control intervention. Others = control interventions that were used in just 1 RCT.

Of the 312 included RCTs, 119 (38.1%) did not define the specific style of yoga used. Thirty-five studies (11.2%) stated that Hatha yoga was used; and 30 others (9.6%) noted that Pranayama or yoga breathing was used, but failing to mention a specific yoga tradition. Of the RCTs that did cite a specific yoga style or approach, Iyengar yoga (31 RCTs, 9.9%), the integrated approach to yoga (16 RCTs, 5.1%) and Sudarshan Kriya yoga (8 RCTs, 2.6%), appeared most often. The remaining 73 trials (23.4%) cited 43 different yoga traditions (Figure 5). Two hundred and forty-four RCTs (78.2%) included yoga postures in their yoga intervention; 232 RCTs (74.4%) included yogic breathing techniques; and 153 RCTs (49.0%) explicitly included meditation (mere relaxation was not counted as meditation). Lectures on yoga philosophy were included by 32 RCTs (10.3%). The reported yoga interventions ranged in length from 1 day (14 RCTs, 4.5%) to 1 year (4 RCT, 1.3%), with a median length of 9 weeks (IQR = 5, 12). Eight (50 RCTs, 16.0%) and twelve (68 RCTs, 21.8%) week programs were by far the most common.Most RCTs compared yoga to one control intervention (or multiple variants of the same control condition), but 62 trials (19.4%) specified two or more different control interventions. In total, 21 different categories of control interventions appeared. More than half of the reported trials cited usual care or no specific treatment as their controls (174 RCTs, 55.8%). Exercise (65 RCTs, 10.1%), other forms of yoga (24 RCTs, 7.7%), and interventions designed to control for the non-specific effects of yoga, such as increased attention by therapists and/or other participants, but without expected specific effects (19 RCTs, 6.1%), were the most commonly used active control conditions (Figure 6).

## Discussion

This bibliometric analysis of the totality of available randomized controlled yoga trials included 312 RCTs from 23 countries, conducted with a total of 22,548 participants. Whilst the earliest of these studies appeared in 1975, the vast majority were post-millennial. The years 2011 and 2012 proved particularly fruitful. Although there was a slight decrease in 2013, the total number of studies published in 2013 is still three times as high as in 2010; suggesting increasing research evidence for yoga in the future. Whether this really marks a trend towards researchers being increasingly intrinsically interested in yoga research or just a policy or funding change remains unclear.

In 2004, an earlier bibliometric analysis of all published yoga trials included 72 RCTs. More than half of these trials originated in India; more than twice as many trials as had then been conducted in the USA [9]. In contrast, the number of Indian and American RCTs in the present analysis was more balanced. Note should be taken, however, of the high number of duplicate Indian yoga publications; suggesting greater than actual Indian research activity. Such duplication increases the risk of falsely overestimating the available evidence for yoga’s effects considerably. This trend towards increased yoga research outside India is important, given previously-voiced concerns about the quality of Indian trials’ research methods and Indian journals’ peer review processes [9]. In addition, Indian yoga trial results may not apply fully to Western societies, as yoga is often seen as a spiritual intervention by Indian participants and as a sport or wellness intervention elsewhere [2, 3]. Systematic reviews also found that Indian yoga trials frequently use extremely time-consuming interventions that may not be feasible in Western participant samples [501]. Accordingly, all four trials with a 1-year intervention period included in this analysis were conducted in India. Trials conducted in the USA or Europe may apply more fully to participants from these cultural backgrounds, as a result.

The analyzed studies had a median of 59 participants, most of them female adults. About a third of these studies were conducted with healthy participants, or those not selected on the basis of their health status. The most commonly included conditions in the remaining RCTs were breast cancer, asthma, depression, type 2 diabetes mellitus, low back pain and hypertension. While this bibliometric analysis does not aim to gauge yoga’s effectiveness as an intervention it seems important to systematically assess the level of evidence provided by these RCTs. Accordingly, a recent meta-analysis included all available 14 RCTs on yoga for asthma. This meta-analysis found short-term beneficial effects on asthma symptoms and pulmonary function [502]. Although not all RCTs available to date were included, systematic reviews and meta-analyses also exist for breast cancer [503, 504], depression [505, 506], type 2 diabetes [507, 508], chronic low back pain [509, 510], hypertension [511–513], pregnancy [514], schizophrenia [515], menopausal symptoms [501, 516], multiple sclerosis [517], and others but not all of the more frequently studied conditions. E.g. to the best of our knowledge, no systematic review on yoga for overweight/obesity is available today. Other RCTs have investigated yoga’s effects in healthy participants, with some comparing different yoga forms [157, 279, 348, 379, 449, 450], the immediate effects of single yoga interventions [279, 379, 456] or the mechanisms by which yoga interventions might work [438]. Such RCTs may yield useful information about yoga’s effects and potential mechanisms also for patients with specific medical conditions.

This bibliometric analysis found that the available research evidence has continuously increased in the past years, especially in recent years, but more research is clearly needed. Whilst 26 medical conditions have appeared in one or two RCTs to date, only six have appeared in ten or more. Indeed, only 17, 14, and 14 RCTs have focused on breast cancer, asthma, and depression, respectively, the most commonly-studied conditions. Despite the fact that many of these studies have found positive effects, yoga research clearly remains limited for most conditions. Besides primary research, up-to-date systematic reviews and meta-analyses are needed at least for the most commonly studied conditions in order to evaluate the level of evidence and strength of recommendation for or against the use of yoga in each condition.

More than 40 different yoga styles were used in the analyzed RCTs. Whilst most trials included yoga postures and breathing, yoga meditation and philosophy were less often used. Yoga is, by definition, a multimodal practice [2, 3]. Although exercise is now often seen as yoga’s main component in Western society, meditation, breathing and lifestyle advice are all traditionally accounted at least equally important [3]. In gauging yoga’s effectiveness, it is important to note that studied interventions can range from the purely meditative to the purely physical in nature. The effects of such diverse interventions are hardly directly comparable, making research to determine the best balance for different medical conditions valuable.

Yoga is not a standardized intervention, nor is it likely (or arguably desirable) that it should become one. This diversity makes it challenging to convey the nature of ‘best’ practice for inclusion in medical guidelines or patient recommendations. The extent to which recent guidelines for designing yoga interventions [518] and control conditions [519] in clinical trials will increase the homogeneity of future research remains unclear. The above evidence suggests that future research might usefully explore the separate effects of yoga postures, breath control, meditation and lifestyle advice, to determine the best interventions for different medical conditions.

This bibliometric analysis has a number of limitations. Firstly, despite the rigorous literature search conducted, it is likely that some RCTs have been missed. Several Indian journals and in particular yoga specialty journals might not be indexed even in Indian medical databases [9]. Secondly, as yoga encompasses a wide variety of practices, borders are blurred between yoga and other, similar, interventions. For example, transcendental meditation is usually seen as distinct from yoga, but is actually based on yoga principles [520]. In the same way, mindfulness-based stress reduction uses yoga postures, but is not commonly seen as a yoga intervention [521]. The exclusion of such interventions from this analysis may, thus, be seen as arbitrary. Thirdly, outcome measures and length of follow-up for outcome assessment were not assessed in this analysis. Finally, this review did not evaluate yoga’s effectiveness or the included trials’ methodological quality. The mere existence of RCTs on a specific condition should not be misinterpreted as evidence of effectiveness in this condition. An in depth study of the located RCTs and – if available – systematic reviews on the effectiveness of these RCTs in a specific condition are necessary to judge the therapeutic value of yoga in this condition. An important next step would be to determine the methodological quality, i.e. risk of bias, nature of outcomes, etc., of the current body of RCTs.

Future yoga research should focus on investigating yoga’s efficacy and safety in conditions that have a major impact on society. Multiple RCTs on the same condition are needed in order to be able to conclusively evaluate its efficacy in this patient population. While it might sometimes be useful to separately publish multiple subanalyses or reanalyses on the same RCT, simply splitting up outcomes of a single RCT to several publications can be regarded as a violation of research ethics – especially if this practice is not disclosed in the respective publications. If a duplicate publication is considered necessary, the authors should disclose and justify this practice in all of the respective publications. Beyond further RCTs, up-to-date systematic reviews and meta-analyses are needed that consolidate the evidence of single RCTs. These reviews should strive to include the totality of available RCTs on a given condition; and probably also an assessment of whether the included RCTs meet recent guidelines for conducting yoga research [518, 519].

## Conclusion

This bibliometric analysis presents the most complete up-to-date review of the randomized controlled yoga trials published to date, and can serve to inform patients, therapists, and researchers on the available yoga research evidence. The results show a marked rise in the number of such trials in recent years; based on research increasingly conducted outside India. For almost four decades, researchers have compared a range of diverse yoga interventions and control conditions, in varied participant samples. This research has had its limitations, with most trials being relatively small in size and failing to explore even common medical conditions frequently. However, this analysis suggests that yoga-based RCTs will increase, diversify and hopefully flourish in future years.

## Authors’ original submitted files for images

Below are the links to the authors’ original submitted files for images. Authors’ original file for figure 1 Authors’ original file for figure 2 Authors’ original file for figure 3 Authors’ original file for figure 4 Authors’ original file for figure 5 Authors’ original file for figure 6
