# Supplementary material for: Characteristics of randomized controlled trials of yoga: a bibliometric analysis
Source: BMC Complement Altern Med. 2014 Sep 2;14:328. doi: 10.1186/1472-6882-14-328 (PMC4161862; doi:10.1186/1472-6882-14-328)
Supplement: Supplementary file 1 — Authors’ original file for figure 1 [file 12906_2013_1900_MOESM1_ESM.pdf]

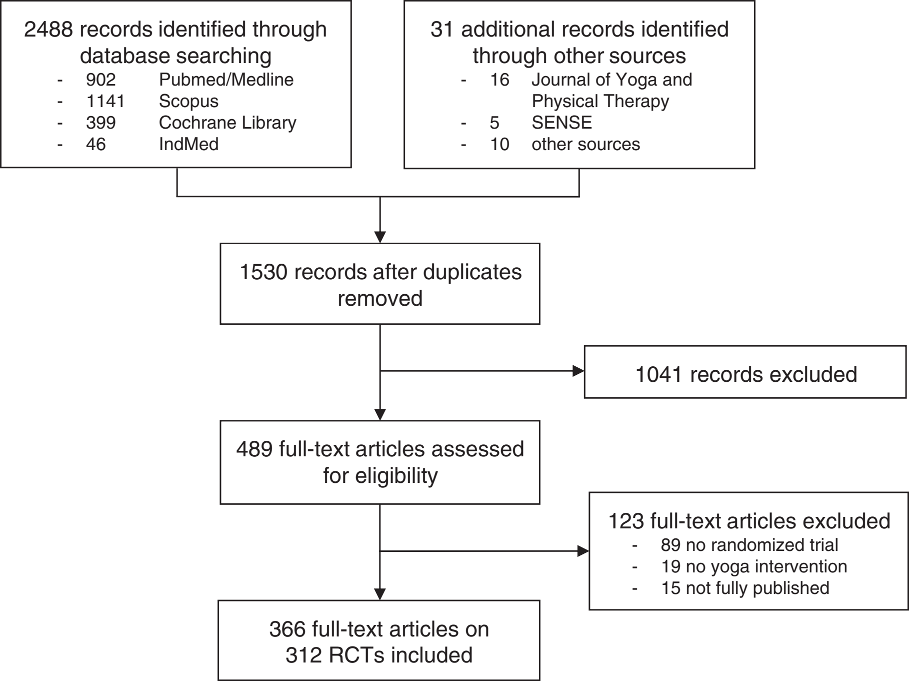
2488 records identified through database searching
- 902 Pubmed/Medline
- 1141 Scopus
- 399 Cochrane Library
- 46 IndMed
31 additional records identified through other sources
- 16 Journal of Yoga and Physical Therapy
- 5 SENSE
- 10 other sources
1530 records after duplicates removed
1041 records excluded
489 full-text articles assessed for eligibility
123 full-text articles excluded
- 89 no randomized trial
- 19 no yoga intervention
- 15 not fully published
366 full-text articles on 312 RCTs included
