# Supplementary figures and images for: Characteristics of randomized controlled trials of yoga: a bibliometric analysis
Source: BMC Complement Altern Med. 2014 Sep 2;14:328. doi: 10.1186/1472-6882-14-328 (PMC4161862; doi:10.1186/1472-6882-14-328)

■ Original publications ■ Duplicate publications

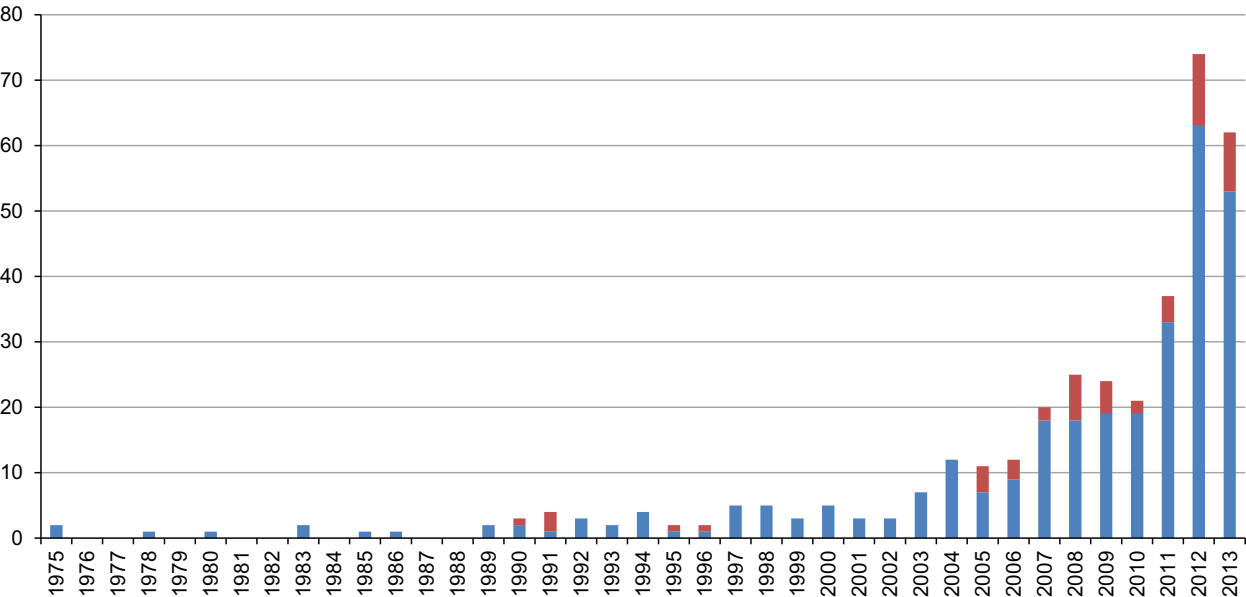

Supplement: Supplementary file 2 — Authors’ original file for figure 2 [file 12906_2013_1900_MOESM2_ESM.pdf]

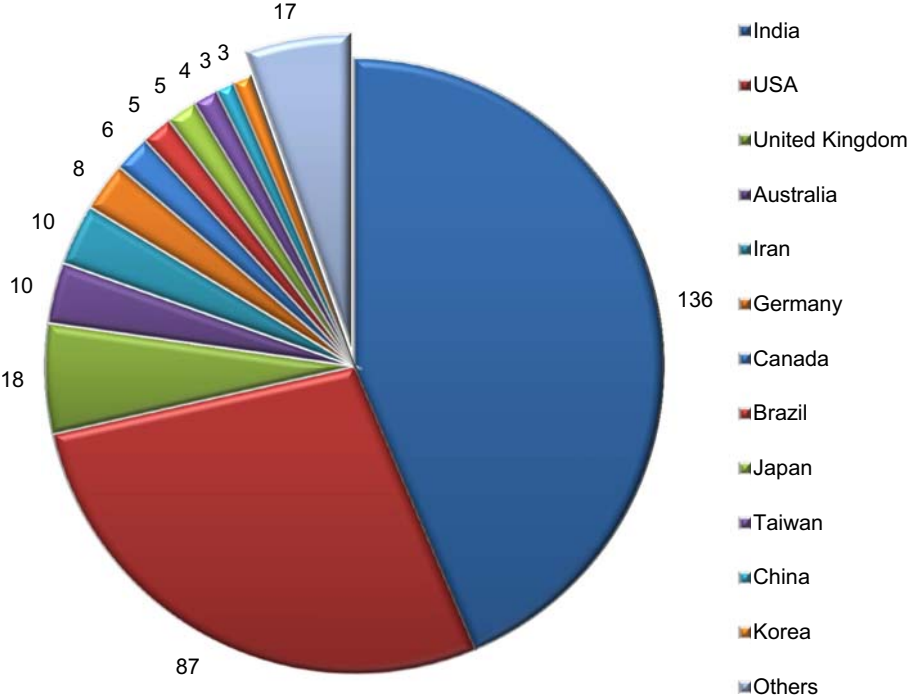

Supplement: Supplementary file 3 — Authors’ original file for figure 3 [file 12906_2013_1900_MOESM3_ESM.pdf]

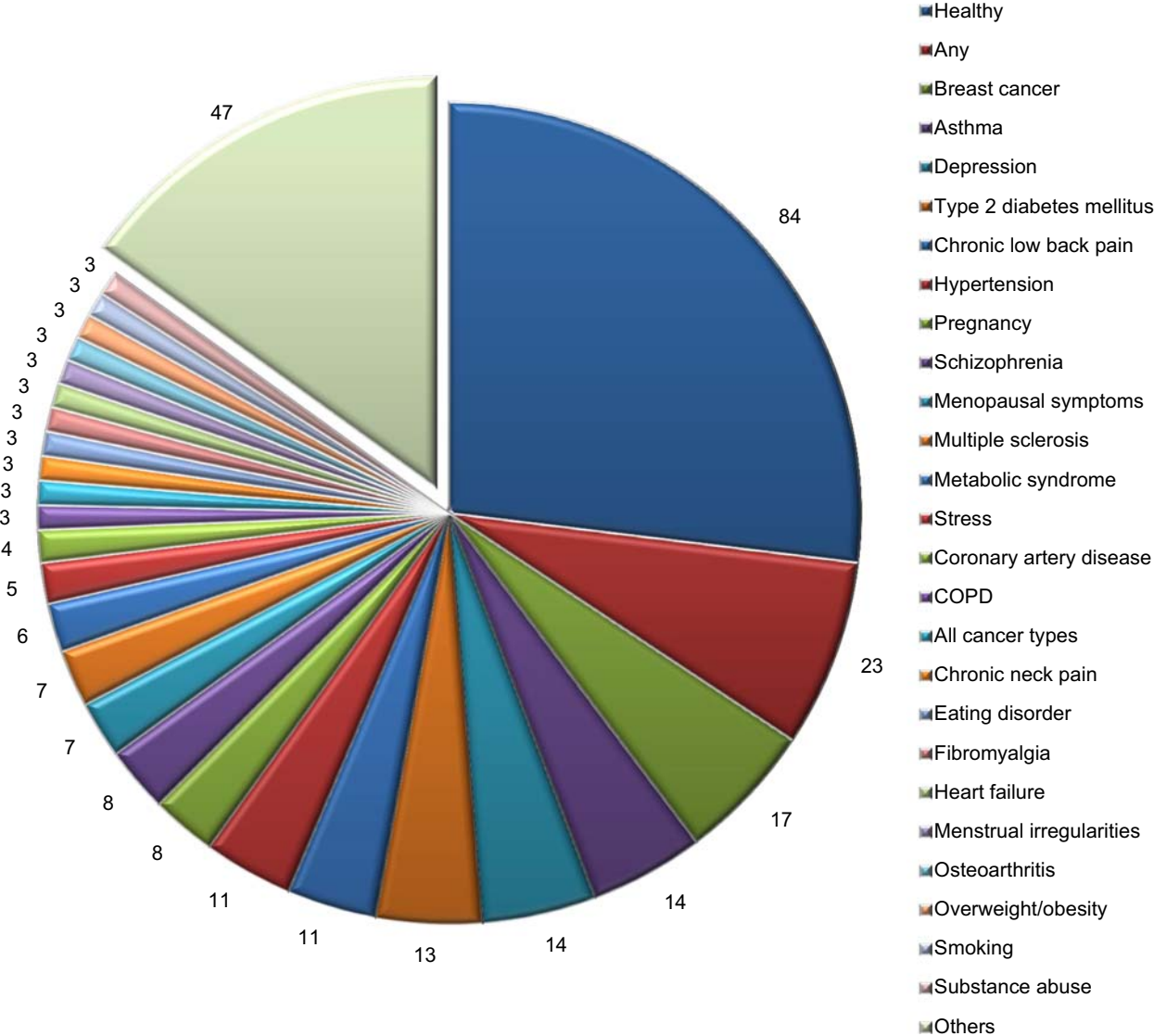

Supplement: Supplementary file 4 — Authors’ original file for figure 4 [file 12906_2013_1900_MOESM4_ESM.pdf]

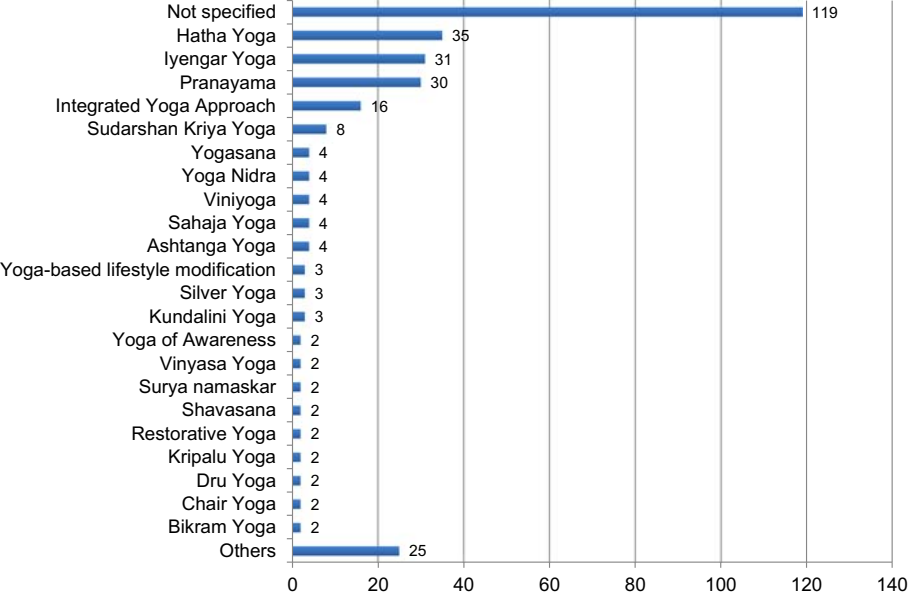

Supplement: Supplementary file 5 — Authors’ original file for figure 5 [file 12906_2013_1900_MOESM5_ESM.pdf]

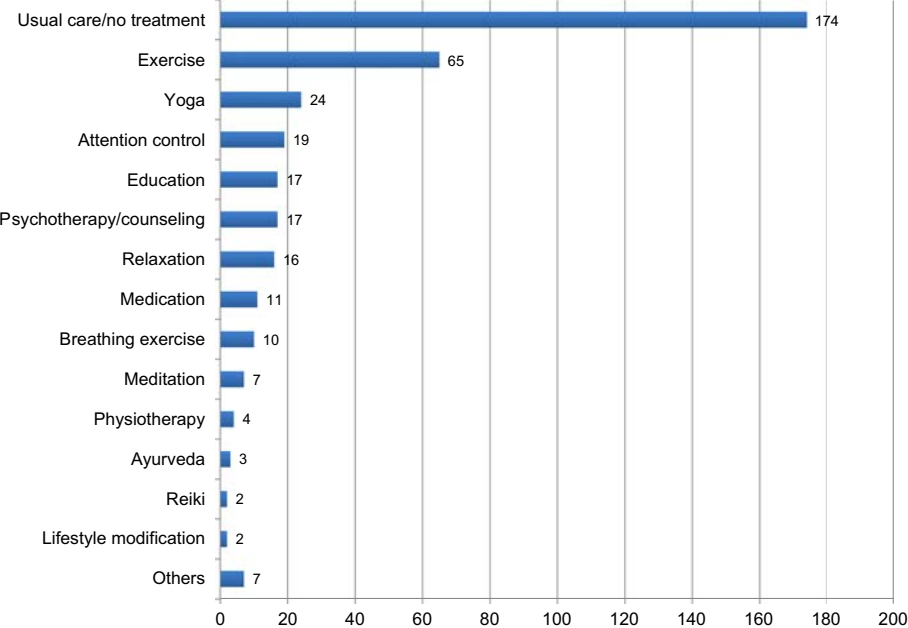

Supplement: Supplementary file 6 — Authors’ original file for figure 6 [file 12906_2013_1900_MOESM6_ESM.pdf]
